# Effect of Hydrogen Peroxide on the Surface and Attractiveness of Various Zirconia Implant Materials on Human Osteoblasts: An In Vitro Study

**DOI:** 10.3390/ma16030961

**Published:** 2023-01-20

**Authors:** Taskin Tuna, Martin Wein, Brigitte Altmann, Thorsten Steinberg, Jens Fischer, Wael Att

**Affiliations:** 1 Department of Prosthodontics and Biomaterials, School of Dentistry, RWTH University Aachen, Pauwelsstr. 30, 52062 Aachen, Germany; 2 Division of Oral Biotechnology, Center for Dental Medicine, Medical Center—University of Freiburg, Faculty of Medicine, University of Freiburg, 79106 Freiburg, Germany; 3 G.E.R.N. Research Center for Tissue Replacement, Regeneration & Neogenesis, Department of Prosthetic Dentistry, Medical Center—University of Freiburg, Faculty of Medicine, University of Freiburg, 79106 Freiburg, Germany; 4 Division of Biomaterials and Technology, Clinic for Reconstructive Dentistry University Center for Dental Medicine UZB, University of Basel, 4058 Basel, Switzerland; 5 Department of Prosthodontics, School of Dental Medicine Tufts University, Boston, MA 02111, USA

**Keywords:** dental implant surface, zirconia, hydrogen peroxide, primary human alveolar bone-derived osteoblasts, cell culture, osseointegration

## Abstract

The aim of this in vitro study was to investigate the effect of hydrogen peroxide (H_2_O_2_) on the surface properties of various zirconia-based dental implant materials and the response of human alveolar bone osteoblasts. For this purpose, discs of two zirconia-based materials with smooth and roughened surfaces were immersed in 20% H_2_O_2_ for two hours. Scanning electron and atomic force microscopy showed no topographic changes after H_2_O_2_-treatment. Contact angle measurements (1), X-ray photoelectron spectroscopy (2) and X-ray diffraction (3) indicated that H_2_O_2_-treated surfaces (1) increased in hydrophilicity (*p* < 0.05) and (2) on three surfaces the carbon content decreased (33–60%), while (3) the monoclinic phase increased on all surfaces. Immunofluorescence analysis of the cell area and DNA-quantification and alkaline phosphatase activity revealed no effect of H_2_O_2_-treatment on cell behavior. Proliferation activity was significantly higher on three of the four untreated surfaces, especially on the smooth surfaces (*p* < 0.05). Within the limitations of this study, it can be concluded that exposure of zirconia surfaces to 20% H_2_O_2_ for 2 h increases the wettability of the surfaces, but also seems to increase the monoclinic phase, especially on roughened surfaces, which can be considered detrimental to material stability. Moreover, the H_2_O_2_-treatment has no influence on osteoblast behavior.

## 1. Introduction

Successful implant anchorage depends on the amount of bone directly contacting the implant surface without soft tissue intervention, which is referred to as osseointegration [1]. In addition to titanium, which is accepted as the gold standard for endosseous dental implants [2], implants made of high-strength ceramics such as yttria-stabilized tetragonal zirconia polycrystal (Y-TZP) are considered a promising alternative due to their physical and chemical properties and outstanding biocompatibility [3,4]. In this context, the surface characteristics of implants, such as topographic and physicochemical properties, play an important role in defining their osseointegration capacity [4,5]. While for titanium it is generally accepted that so-called microrough implant surfaces lead to improved tissue and cell response compared to smooth surfaces [6,7,8] and also have a higher percentage of bone-to-implant contact (BIC) and require higher forces to release the implant-bone anchorage [5,7,9,10], for zirconia it is currently still controversial which implant surface is best suited for the aforementioned purposes [11,12]. Compared to contemporary titanium implants, whose surfaces are modified either by turning, acid etching, grit-blasting, coating, oxidizing, or combinations of these techniques [13,14], similar surface modifications are commercially available for zirconia implant surfaces [3,4,15]. However, these modification procedures are considered more complicated for zirconia for two reasons: on the one hand, because of the difficulties in processing and manipulating its hard surface with respect to ablation techniques and because of the strong chemical bonds with respect to etching techniques [2,16,17]. On the other hand, the surface treatment procedures described above, or even strong heat exposure, can cause surface and subsurface micro-cracks, which can lead to a deterioration of the fatigue behavior due to phase transformation from tetragonal to monoclinic, increasing the material’s susceptibility to faster moisture-assisted transformation, i.e., low-temperature degradation (LTD) with unfavorable long-term clinical behavior as a consequence [2,3,18]. Thus, at present, there is no consensus on the surface treatment and associated morphological aspects of zirconia surfaces to improve osseointegration [2,3,4,19]. However, in order to accelerate the osseointegration process, surface modifications are recommended which, for example, increase the wettability and surface energy to which osteoblasts can adhere, spread and proliferate. [3,18]. Thus, in recent years, research has been conducted into alternative ways of bioactivating surfaces by chemical and topographic modifications of dental implants. These include surface treatments such as coatings with biologically active materials (bioceramics, ions and biomolecules) [13,20,21] but also treatment with UV light alone. In the latter, several groups of authors reported that irradiation of machined and roughened surfaces of zirconia and titanium with UV light significantly improves the osteoblast response. [22,23,24]. The improved bioactivity on both materials was associated with the photochemical and photocatalytic removal of surface carbons, due to their properties as semiconducting photocatalytic materials [24,25,26].

Similar to UV treatment, the application of hydrogen peroxide (H_2_O_2_) is considered a very efficient decontamination method due to its oxidation ability, which occurs through spontaneous and exothermic decomposition in water and oxygen [27,28]. The decomposition reaction releases highly reactive oxidative species (ROS) that can oxidize or remove a variety of organic and inorganic structures [27,28]. Thus, it is likely that H_2_O_2_ decomposes hydrocarbons on the surface through an oxidative reaction, so surface decontamination by H_2_O_2_ could be similar to UV treatment. In this context, the question arises as to how the treatment of implant surfaces with H_2_O_2_ affects their surface chemistry and whether the treatment with H_2_O_2_ makes them more attractive for the attachment of osteoblast cells. In this regard, the effect of pretreatment with H_2_O_2_ alone or in combination with other media on titanium implant surfaces has already been investigated in previous studies, and a positive influence on protein resorption and cell behavior has been reported [29,30]. As far as the authors are aware, there is only one study on the effect of H_2_O_2_ on zirconia surfaces. However, this one focused on the effect of home bleaching solutions (10% carbamide peroxide and 6% H_2_O_2_) on the optical properties of monolithic zirconia used for dental restorations and found that home bleaching agents can affect both translucency and color [31].

Against this background, the aim of this study was to investigate the physicochemical effect of H_2_O_2_-treatment on the surface of two different zirconia-based implant materials with different surface topographies and the response of human alveolar bone-derived osteoblasts (AO) to these surfaces.

## 2. Materials and Methods

### 2.1. Zirconia Samples and H_2_O_2_-Treatment

For the present study, we used ceramic discs (20 mm diameter and 1.5 mm thickness) of two zirconia-based core materials with smooth (Zr1m and Zr2m) or rough (Zr1r and Zr2r) surfaces. We investigated identical platelet samples with the same analytical methods of two previous studies on the effect of UV light on the surface structure and attractiveness of human osteoblasts [24,32]. The following description of the materials and methods used is based on the cited publications and therefore kept short.

Material Zr1 is an experimental platelet-reinforced zirconia consisting of the metal oxides ZrO_2_ (85.7 wt%), Al_2_O_3_ (8.3 wt%), Y_2_O_3_ (4,3 wt%) and La_2_O_3_ (1.7 wt%). The Zr2 material is a conventional yttrium tetragonally stabilized zirconia (Y-TZP) containing 93 wt% ZrO_2_, 5 wt% Y_2_O_3_, 1.9 wt% HfO_2_ and 0.1 wt% Al_2_O_3_. All test specimens were provided by the material supplier (CeramTec, Plochingen, Germany). Production was carried out by cutting the discs from a rod in the white state using a computer-controlled machine and a carbide tool. The blanks were densely sintered and hipped. While the smooth surface of Zr1 corresponded the sintered material, the smooth surface of Zr2 was additionally polished with 3µm diamond paste. To roughen the discs, Al_2_O_3_ at a blasting pressure of 6 bar and a grain size of the abrasive of 105 µm was used. After blasting, acid etching in 38–40% hydrofluoric acid (HF) was performed for one hour, followed by thermal treatment at 1250 °C under an oxidative atmosphere with a three-hour dwell time. [33].

Prior to testing, all specimens were cleaned with 70% ethanol and double distilled water (ddH_2_O), followed by ultrasonic cleaning in ddH_2_O for 5 min and air-drying. Samples were then sterilized by low-temperature hydrogen peroxide gas plasma sterilization at 55 °C (STERRAD^®^, 100NX™ System, Johnson & Johnson Medical, Norderstedt, Germany) followed by sealing and storage in the dark for one month [15]. For H_2_O_2_ surface treatment, discs were immersed in a bath containing 20% H_2_O_2_ for 2 h. Subsequently, the samples were rinsed three times with ddH_2_O and air-dried under sterile conditions.

### 2.2. Material Surface Analysis

The surface morphology of the H_2_O_2_-treated and untreated zirconia discs was investigated by scanning electron microscopy (SEM) (LEO 1525 Field Emission Gun, FEG SEM, Zeiss, Jena, Germany). Atomic force microscopy (AFM) (Nanoscope IIIa, Veeco-Digital Instruments, Santa Barbara, USA) was used to investigate the surface roughness of each sample. For this purpose, nine different areas per sample were measured (n = 9 per material group). To evaluate the surface wettability, the contact angle (CA) of H_2_O_2_-treated and untreated zirconia discs was measured. The contact angles of four 1-µL H_2_O droplets on a single disc of each material (n = 4) were determined (Dataphysics GmbH, Filderstadt, Germany). To examine the chemical composition of the material surfaces before and after H_2_O_2_-treatment, electron spectroscopy for chemical analysis (ESCA) was performed using X-ray photoelectron spectroscopy (XPS) (Perkin Elmer PHI 5600 ESCA System, Physical Electronics, Inc., Chanhassen, Minnesota, USA) under ultrahigh vacuum conditions (5 × 10^−8^ mbar). The changes in chemical composition were quantified using data analysis software for XPS (MultiPak version 9, Physical Electronics, Inc., Chanhassen, Minnesota, USA). In order to examine the surface stability of the zirconia samples, an X-ray diffractometer (XRD) with a Cu-Kα source (D8 Advance X-ray diffractometer, Bruker AXS, Karlsruhe, Germany) was used to determine the crystalline phases before and after H_2_O_2_-treatment of the surfaces. XRD spectra were collected on the different samples over a 2θ range between 15° and 70° at a step size of 0.01° and 4s per step. The monoclinic phase fraction was calculated by the Garvie-Nicholson method [34]. The identification and correction of peaks (monoclinic/tetragonal/cubic) based on the ICSD database [35].

### 2.3. Isolation and Cultivation of Primary Human Osteoblasts

Primary human alveolar osteoblasts (AO) were obtained from alveolar bone explants of a healthy adult patient (male, 42 years) during implantation as described in a previous publication [36]. For this purpose, the ethics committee of the Albert Ludwigs University of Freiburg had approved the harvesting of oral bone explants (approval number 411/08).

The cultivation of osteoblasts obtained from alveolar bone fragments was performed in Dulbecco’s Modified Eagle’s Medium (Low Glucose (1 g/L) (DMEM, PAA Laboratories, Coelbe, Germany), supplemented with 1% glutamine (GlutaMax^TM^, Life Technologies, Darmstadt, Germany), 10% fetal calf serum (Biochrom AG, Berlin, Germany), 10 mM Na-ß-glycerophosphate, 50 μg/mL ascorbic acid, 0.1 μM dexamethasone and 0.2% kanamycin (Sigma-Aldrich, Steinheim, Germany).

Cells were maintained in a humidified 37 °C incubator with 5% CO_2_. At 80% confluence, cells were separated with 0.1% trypsin/0.04% EDTA (Biochrom AG, Berlin, Germany) and seeded at a concentration of 3 × 10^4^ cells/cm^2^ on zirconia discs that were either pretreated in a 20% H_2_O_2_ bath for 2 h or left untreated. The culture medium was changed every three days, and all experiments were run with passage 5 and passage 6 osteoblasts.

### 2.4. Analysis of the Initial Cell Attachment and Cell Area

Initial attachment and cell area of AO on H_2_O_2_-treated and untreated samples were studied by fluorescence microscopy (Biozero, BZ-9000, Keyence, Neu-Isenburg, Germany) of fluorescent phalloidin-labeled actin and DNA quantification.

For phalloidin labeling of the actin cytoskeleton of the osteoblasts, adherent cells on zirconia discs were first rinsed with PBS buffer and fixed in 3.8% formalin (Carl Roth, Karlsruhe, Germany) after 4 h and 24 h of culture. Then, samples were treated with 0.02% TritonX-100 (Sigma-Aldrich, Taufkirchen, Germany) in PBS for 5 min and then incubated with green fluorescent Alexa 488/phalloidin (1:40, Life Technologies, Darmstadt, Germany) for 40 min. The nuclei were stained with DAPI nucleic acid stain (Life Technologies, Darmstadt, Germany) for 15 min. Subsequently, images of representative areas of the cultured samples were photographed in order to assess cell morphology and cell size. For the latter, cell size of 27 randomly sampled cells from different disc areas from three discs each (n = 81) was quantified once at 4 h and once at 24 h after seeding for H_2_O_2_-treated and untreated surfaces using the integrated image analysis tool of the fluorescence microscope (Biozero, BZ-9000, Keyence, Neu-Isenburg, Germany).

The quantity of initially attached cells on H_2_O_2_-treated and untreated zirconia discs was investigated indirectly by determining the DNA content of cells that had successfully attached to the surfaces after 4 h and 24 h of culture. For this purpose, 5 × 10^4^ cells were seeded and cultivated for the already mentioned duration on the discs, and then attached cells were washed once with PBS and lysed by a freeze-thaw cycle at −80 °C in 400 μL TE buffer (10 mM Tris-HCl, 1mM EDTA, pH7.5). DNA quantification was performed using the Quant-iT^TM^ PicoGreen assay (Invitrogen, Karlsruhe, Germany) according to the manufacturer’s instructions.

### 2.5. Cell Proliferation Assay

For quantitatively measuring the cell proliferation activity (cell growth and cell division), the cell viability assay “alamarBlue^®^ Metabolic Assay” (AbD Serotec, Düsseldorf, Germany) was used. The assay was carried out on days 3, 7 and 14 of culture by substituting the culture medium with alamarBlue^®^ reagent. The supernatant was analyzed after 2 h at 37 °C by measuring fluorescence according to the manufacturer’s instructions. The percentage of alamarBlue^®^ reduction in the samples was calculated using a 100% reduced alamarBlue^®^ control as a reference, prepared according to the manufacturer’s protocol.

### 2.6. ALP Activity of Osteoblasts

The capacity of AO to form mineralized extracellular matrix on the zirconia-based surfaces was examined by determining the alkaline phosphatase (ALP) activity in cell lysates (QuantiChrom™ ALP Kit, BioAssay Systems, Hayward, CA, USA). This method uses p-nitrophenyl phosphate, which is hydrolyzed by ALP to a yellow-colored product. The rate of the reaction is directly proportional to the enzyme activity, and the enzyme ALP is an important regulator of bone formation due to its promotion of mineralization processes [37]. The ALP assay was performed after 7 and 14 days of culture on untreated and H_2_O_2_-treated surfaces according to the manufacturer’s protocol. The adherent cells on the zirconia discs were washed twice with PBS buffer, lysed with 500 µL Complete Lysis-M Buffer (Roche, Mannheim, Germany) for 10 min at room temperature and stored at −20 °C. After thawing the cell lysate, protein concentration in all samples was quantified using the Pierce^®^ 660 nm Protein Assay (Thermo, Dreiech, Germany). Alkaline phosphatase activity was determined according to the manufacturer’s protocol and normalized to 5 µg total protein for each reaction.

### 2.7. Statistical Analysis

The cell culture experiments for the analysis of the DNA content, cell proliferation (alamarBlue assay) and ALP activity were performed in triplicates in three independent experiments (n = 9). Data of the cell culture experiments and the surface roughness parameters of each material were compared for statistically significant differences by using the Student’s t-test with a significance level of *p* < 0.05. For contact angle measurements, the statistical package R (R CRAN, Version 4.3) was used for statistical analyses. Exact Mann-Whitney tests were calculated (R-library “coin”) to show between-treatment-group differences in continuous data. A *p*-value below 0.05 (*p* ≤ 0.05) was considered a significant difference.

## 3. Results

### 3.1. Surface Morphology and Hydrophilic Properties of H_2_O_2_-Treated and Untreated Zirconia Surfaces

**Figure 1:**
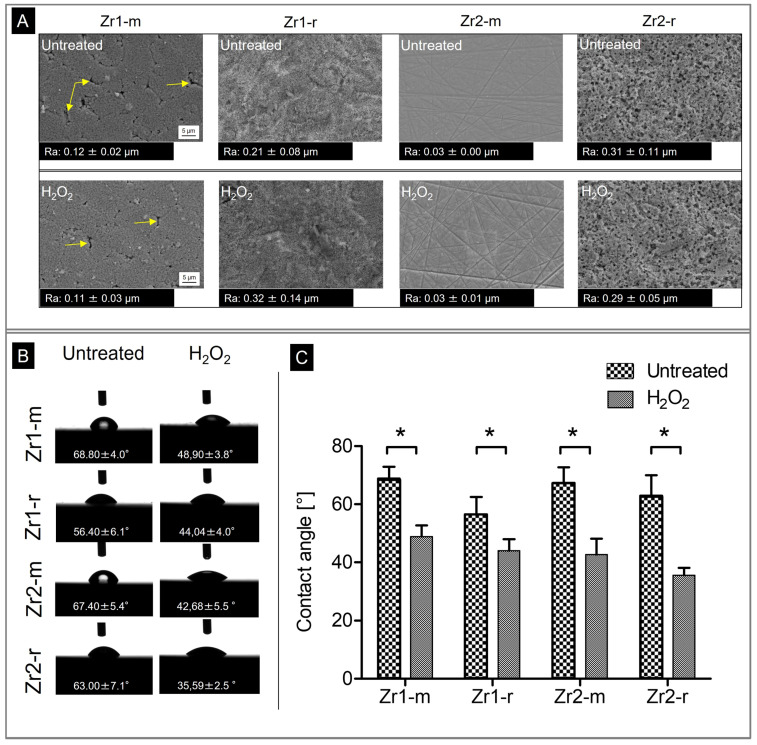
(**A**–**C**) Morphology of the zirconia-based surfaces before and after H_2_O_2_-treatment and changes in their hydrophilic properties after H_2_O_2_-treatment. (**A**) SEM images at 5000× magnification of the four untreated and H_2_O_2_-treated surfaces Zr1-m/r and Zr2-m/r (mean average roughness data ± SD (Ra in µm) given below SEM images). (**B**) Representative photographic images of contact angle measurements of 1 µL water droplets pipetted onto zirconia discs with and without H_2_O_2_-treatment for 2 h (mean contact angles ± are embedded in the contact angle images). (**C**) Comparison of the contact angle development with and without H_2_O_2_-treatment between the four different samples. Statistical significances with *p* < 0.05 are labeled with “*” in the graphs.

In order to characterize the surface morphology and surface roughness of the different zirconia-based materials under study, we performed SEM and AFM analysis. The assessment by SEM showed no significant changes in the surface properties of each material after H_2_O_2_-treatment. As presented in Figure 1A, all four zirconia specimens differed in terms of their surface structure. In detail, Zr1-m had a flat surface with homogenous sub-micron porosity and a high number of fine grain-like pores in a range of 50–100 nm in size. In addition, single large pores with 1 × 5 µm in size were distributed irregularly on the surface (Figure 1A, yellow arrows). In contrast, Zr2-m showed a relatively smooth and non-porous surface with superficial polishing tracks. No pores and no grainy structures were visible. The rough surface Zr1-r appeared very compact with little dimples and pores. In contrast, Zr2-r exhibited a very rough surface with greater porosity and waviness than Zr1-r.

Quantitative analysis of the surface roughness by AFM exhibited significantly different surface roughness values between all four material groups with Ra values ranging from 0.03 ± 0.00 µm to 0.32 ± 0.14 µm µm (*p* < 0.05; n = 9) (Figure 1A). H_2_O_2-_treatment did not cause any significant changes in the surface roughness of the different samples (*p* > 0.05). The examination of the surface wettability showed similar average contact angles for all untreated surfaces ranging from 56.4 ± 6.1° to 68.8 ± 4.0° (*p* > 0.05; n = 4) (Figure 1B). Since the contact angle was below 90° for all surfaces, they could be classified as hydrophilic [38]. Surface treatment with H_2_O_2_ resulted in a significant increase in the wettability of the surfaces compared to the corresponding untreated controls, as evidenced by lower contact angle values. In detail, the contact angles decreased on Zr1-m from 68.8 ± 4.0° to 48.9 ± 3.8 (*p* < 0.001), on Zr1-r from 56 ± 6.1 to 44.04 ± 4.0 (*p* = 0.001), on Zr2-m from 67.4 ± 5.4 to 42.68 ± 5.5 (*p* < 0.001) and on Zr2-r from 63.00 ± 7.1 to 35.59 ± 2.5 (*p* < 0.001) (Figure 1B,C).

### 3.2. Chemical Surface Composition of H_2_O_2_-Treated and Untreated Zirconia Surfaces

**Figure 2:**
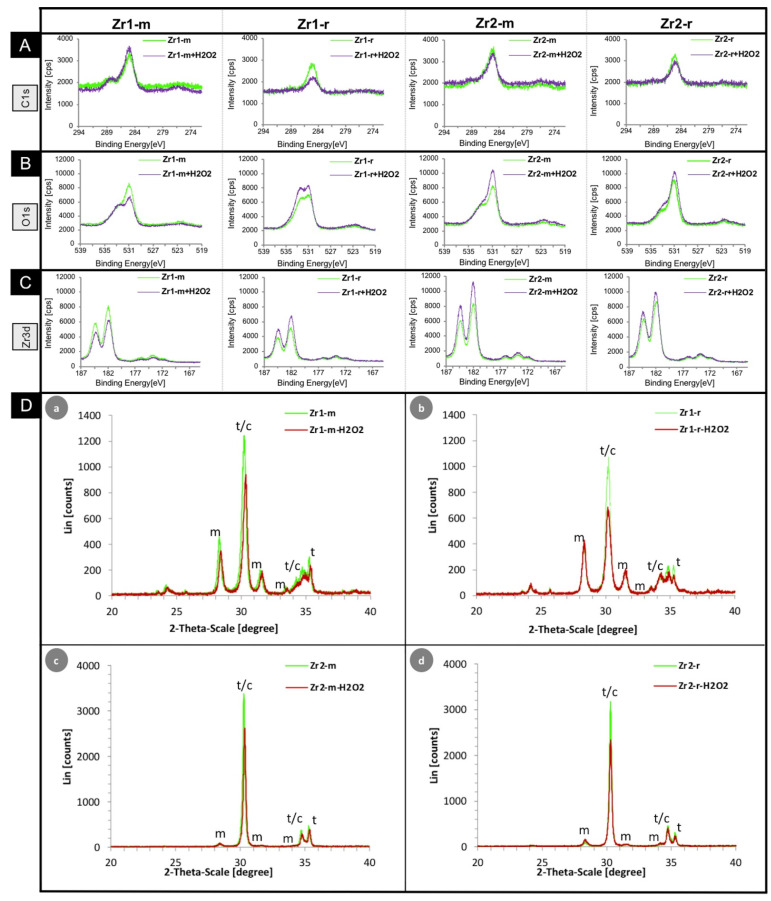
(**A**–**D**) XPS detail spectra of the smooth and roughened surfaces of the two zirconia materials (Zr1-m/r and Zr2-m/r) and changes in the chemical composition after H_2_O_2_-treatment for the following electrons: (**A**) = C1s; (**B**) = O1s; (**C**) = Zr3d. (**D**) XRD graphs of the four surfaces (a/b: Zr1-m/r and c/d: Zr2-m/r) (t = tetragonal ZrO_2_, m = monoclinic ZrO_2_; c = cubic phase). Typical peaks of the monoclinic ZrO_2_ phase at 2θ of 28.3° and 31.5° and peaks of the tetragonal/cubic phase at 2θ of 30.3° were detected on all surfaces. Additional specific peaks for the tetragonal phase could be identified, but they overlapped the cubic phase peaks. The surface of the Zr1 material showed several additional peaks indicative of additional crystalline structures.

The analysis of the chemical surface composition by XPS confirmed the elemental components of the two ceramic materials, which were mentioned in Section 2.1. For more information about the basic surface chemistry of both materials, see Tuna et al. [32]. The XPS detail spectra of the smooth and roughened surfaces of the two materials and their changes after H_2_O_2_-treatment for the C1s, O1s, and Zr3d electrons are presented in Figure 2A–C.

The XPS investigation showed a decrease in surface carbon (C1s/285 eV) at the three surfaces Zr1-r (−60%), Zr2-m (−33%) and Zr2-r (−46%), while the carbon content at surface Zr1-m increased by 32%. The opposite was observed for the oxygen (O1s/531 eV) and zircon (Zr3d/182 eV) contents. The oxygen and zircon contents at the three material surfaces Zr1-r (O1s: +26%; Zr3d: +25%), Zr2-m (O1s: +10%; Zr3d: +35%) and Zr2-r (O1s: +19%; Zr3d: +14%) increased, while the oxygen and zircon contents at Zr1-m (O1s: −14%; Zr3d: −22%) decreased (Table 1).

### 3.3. Crystalline Phases of H_2_O_2_-Treated and Untreated Zirconia Surfaces

To get an insight into the surface stability of the zirconia samples before and after H_2_O_2_-treatment, the crystalline phases of the untreated and H_2_O_2_-treated samples were determined by XRD method. The scans of the four surfaces are shown in Figure 2D, and the development of the percentages of the monoclinic phase of all four surfaces without and with H_2_O_2_-treatment are presented in Table 2.

XRD analysis revealed a different content of crystal phases between the smooth and the roughened surfaces within the same material and a general increase in monoclinic phase at all samples after H_2_O_2_-treatment (Table 2). While both untreated surfaces of material Zr2 showed only a small amount of monoclinic phase with Zr2-m ≈ 3.4 wt.% and Zr2-r ≈ 4.9 wt.%, a 7–10 times higher monoclinic phase was observed on the untreated Zr1 materials with Zr1-m ≈ 33.6 wt.% and Zr1-r ≈ 34.5 wt.%.

After the application of H_2_O_2_, an increase in monoclinic phase was observed on all zirconia surfaces (Table 2). An increase in monoclinic phase is generally rather undesirable in zirconia ceramics since it can lead to a weakening of the surface stability [39].

### 3.4. Cell Attachment of Osteoblasts on H_2_O_2_-Treated and Untreated Zirconia Surfaces

The examination of the initial osteoblast attachment and the morphometrical quantification of the extent of cell spreading on the test surfaces was performed by fluorescence-based labeling of the actin cytoskeleton at 4 h and 24 h after cell seeding.

**Figure 3:**
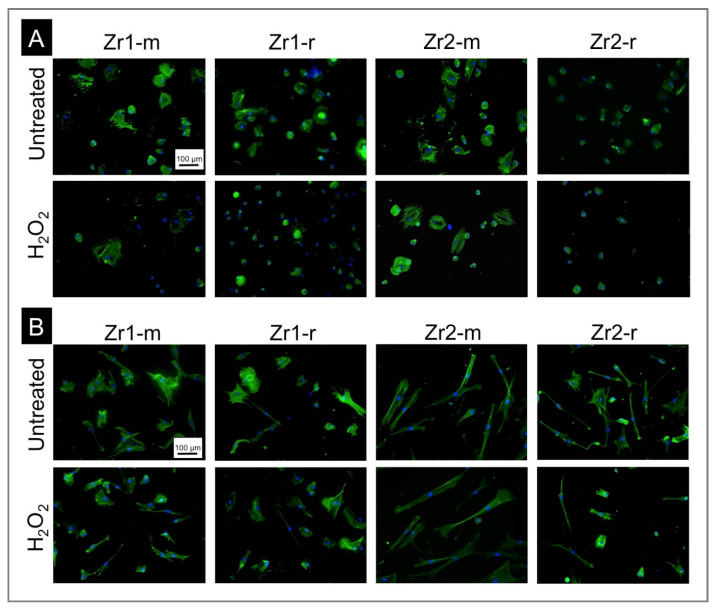
(**A**,**B**) Initial attachment and spread of AO 4 h (**A**) and 24 h (**B**) after seeding onto untreated and H_2_O_2_-treated zirconia surfaces. Representative fluorescence microscopy images of cell cultures with dual staining of DAPI for nuclei (blue) and phalloidin for actin filaments (green) are shown.

According to the fluorescence microscopy images in Figure 3A, in the early phase at 4 h after cell seeding, osteoblasts had a round shape and had little cytoskeletal development on all material surfaces under study. The cells on both smooth surfaces Zr1-m and Zr2-m appeared similar on both untreated and H_2_O_2_-treated surfaces. Cells on the rough surface Zr2-r also appeared similar after H_2_O_2_-treatment but looked generally smaller than cells on Zr2-m surfaces. The cells attached on rough Zr1-r appeared to have spread more on untreated surfaces when compared to the corresponding H_2_O_2_-treated surface. After 24 h, the cells had spread on all surfaces (Figure 3B). The differences in terms of cell spreading that were visible after 4 h on the rough Zr1-r versus matched H_2_O_2_-treated surfaces were thereby no longer detectable. The cells on the untreated rough Zr2-r surface appeared to have spread more than on the H_2_O_2_-treated surface. Between the untreated and H_2_O_2_-treated smooth surfaces, no difference between the cells was visible.

**Figure 4:**
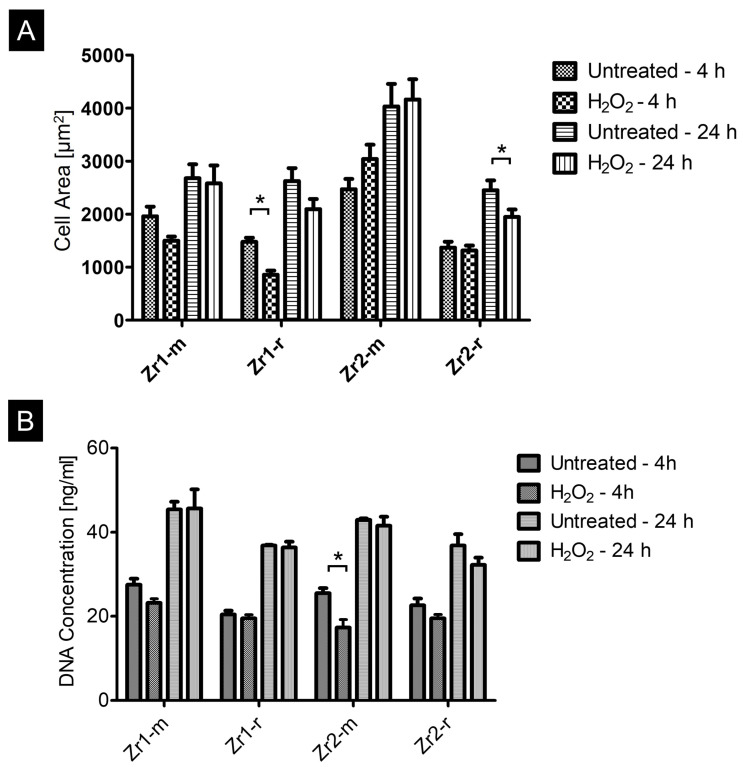
(**A**,**B**) Comparison of the cell area (**A**) and the DNA concentration (**B**) on untreated and H_2_O_2_-treated zirconia surfaces after 4 h and 24 h of culture. The image analyzer tool of the fluorescence microscope was used to perform cell morphometric measurements. Data are mean ± SD (n = 81) evaluated from 27 cells from each disc. Statistical significances with *p* < 0.05 are labeled with “*” in the graphs.

The quantitative morphometric analysis of the cell area revealed that osteoblasts on Zr1-r displayed lower cell area values than on corresponding untreated surfaces at 4 h (*p* < 0.05), and that osteoblasts on Zr2-r showed lower cell area values on H_2_O_2_-treated surfaces compared to untreated surfaces after 24 h (*p* < 0.05). No significant differences were detected within the other groups (*p* > 0.05) (Figure 4A).

With respect to the number of attached cells, the DNA quantification demonstrated a lower attachment rate on H_2_O_2_-treated Zr2-m when compared to the corresponding untreated surfaces at 4 h (*p* < 0.05) (Figure 4B). This difference in DNA content and thus cell amount on the untreated versus H_2_O_2_-treated surfaces was, however, no longer observable at 24 h. A similar trend, albeit not statistically significant, was observable for Zr1-m and Zr2-r at 4 h, and Zr2-r at 24 h (Figure 4B).

In summary, the presented data concerning morphogenesis and cell amount on the test surfaces indicate that the H_2_O_2_-treatment had no marked influence on osteoblast attachment.

### 3.5. Proliferation of Osteoblasts on H_2_O_2_-Treated and Untreated Zirconia Surfaces

In order to examine the influence of H_2_O_2_-treatment of the zirconia-based materials on cell proliferation, the metabolic activity of osteoblasts was determined by the alamarBlue assay at days 3, 7 and 14. The alamarBlue reporter dye is metabolized in the mitochondria of the cells and thus provides information on cell growth.

**Figure 5:**
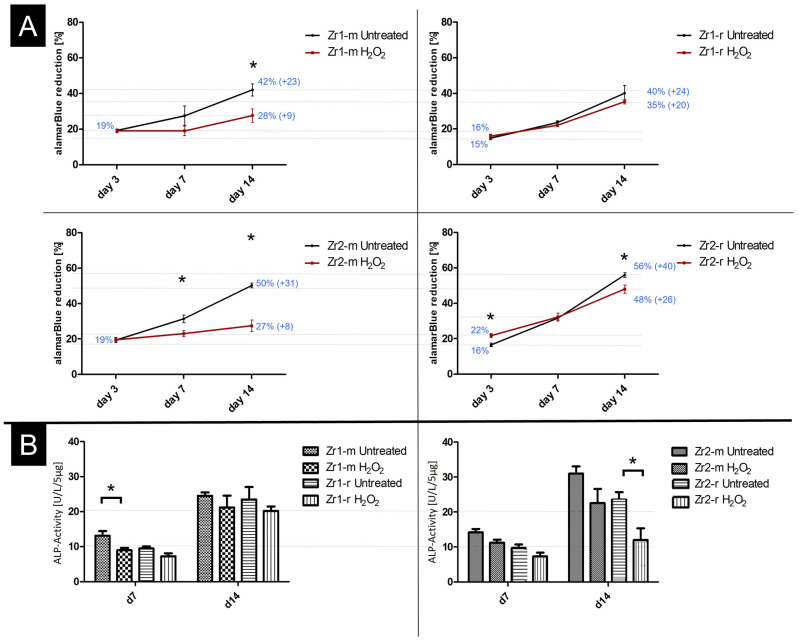
(**A**,**B**) Proliferation and differentiation activity of AO on H_2_O_2_-treated and untreated surfaces. (**A**) Cell proliferation by the alamarBlue**^®^** metabolic assay at 3, 7 and 14 days of culture. For a clear illustration of the development of proliferation rates, the graphs are labeled with the most important percentages at days 3, 7 and 14. (**B**) Determination of differentiation capacity by measuring alkaline phosphatase activity of the normalized protein amount (5 µg protein for each reaction) between H_2_O_2_-treated and untreated groups at days 7 and 14. The data were collected from triplicates of three independent experiments (n = 9), with a predefined constant cell number of 5 × 10^4^ cells per disc. Statistical significances with *p* < 0.05 are labeled with “*” in the graphs.

The alamarBlue (AB) assay revealed a continuous increase in the metabolic reduction of the reporter dye AB and thus indicated that all surfaces under study supported osteoblast proliferation (Figure 5A). By comparing the metabolic activity of the cells on the H_2_O_2_-treated versus untreated surfaces, it becomes apparent that the metabolic activity was superior on untreated smooth Zr1m and Zr2m with matched H_2_O_2_-treated materials at days 7 and 14, and at day 14 even being significant for both smooth surfaces. This effect was, however, less clear on rough Zr1-r and Zr2-r (Figure 5A; compare Zr1-m with Zr1-r and Zr2-m with Zr2-r).

The increase in AB reduction from day 3 to day 14 on smooth surfaces was thereby +23% for Zr1-m vs. +9% for Zr1-m+H_2_O_2_, and +31% for Zr2-m vs. +8% for Zr2-m+H_2_O_2_ (Figure 5A). On rough surfaces, the change in AB reduction from day 3 to day 14 was +24% for Zr1-r vs. +20% for Zr1-r+H_2_O_2_, and +40% for Zr2-r vs. +26% for Zr2-r+H_2_O_2_.

These results point to a surface roughness-dependent effect of H_2_O_2_ on the cell proliferative capacity.

### 3.6. APL Activity of Osteoblasts on H_2_O_2_-Treated and Untreated Zirconia Surfaces

The extracellular matrix mineralization capacity of osteoblasts on the different material surfaces was analyzed by determining the ALP activity at days 7 and 14. The enzyme ALP is an important regulator of bone formation due to its promotion of mineralization processes [37]. Therefore, it is widely used as a marker for early osteoblast differentiation in vitro [40,41].

The results show that the APL activity was significantly lower on H_2_O_2_-treated Zr1-m at day 7 and Zr2-r at day 14 when compared to corresponding untreated surfaces (*p* < 0.05) (Figure 5B). Within the other groups, no significant difference between untreated and H_2_O_2_-treated groups were visible. Thus, except for the two mentioned surfaces, treatment with H_2_O_2_ appears to have no effect on the mineralization capacity of osteoblasts.

## 4. Discussion

This study addressed the effects of H_2_O_2_-treatment of zirconia-based implant materials with different surface properties and the effect on primary human osteoblasts (AO). For this purpose, test discs previously treated with 20% H_2_O_2_ solution for 2 h were used and compared with untreated discs as controls.

The present results show that the exposure of zirconia surfaces to H_2_O_2_ did not alter their topographic appearance and roughness values (Figure 1A), but, in contrast, partially significant changes were observed in the physical and chemical properties. In particular, the wettability of all surfaces increased significantly in the direction of hydrophilicity. While the increase in wettability in a previous study with UV irradiation of the same material surfaces was accompanied by a significant decrease in the carbon content of all surfaces [32], this change was, however, only observed for the three surfaces Zr1-r, Zr2-m and Zr2-r in this study. Here, the carbon content on the Zr1-m surface increased by as much as 32%, although the contact angle of Zr1-m was significantly reduced after H_2_O_2_-treatment, implying an improvement in wettability. As with the other three surfaces, Zr1-r, Zr2-m and Zr2-r, we would have expected a reduction in surface carbon content. A possible explanation for this phenomenon could be the following: Zr1-m exhibited an as-sintered surface, while all the other specimens underwent a surface treatment, which removed the superficial layer, thus revealing the bulk material. As-sintered and mechanically treated surfaces differ in their properties [42]. Assuming disorder and lattice distortion with oxygen, vacancies are more pronounced at the as-sintered surface compared to surfaces exposing the bulk structure, then a higher reactivity toward CO and CO_2_ adsorption may be supposed [43]. With the specimens Zr1-r and Zr2-r, H_2_O_2_-treatment led to a higher monoclinic phase ratio (+37% and +63%, respectively) in comparison to Zr1-m and Zr-2m (+2.4% and +18%, respectively; the increase of Zr2-m from 3.4% to 4% should not be overinterpreted, because at low monoclinic content, the measurement accuracy in XRD is less accurate). This effect indicates that oxygen vacancies are filled by oxygen originating from H_2_O_2_. In contrast, the monoclinic phase ratio at the Zr1-m surface remains nearly constant after H_2_O_2_-treatment, indicating that a stable situation is given. After H_2_O_2_-treatment, the Zr1-m surface may be cleaned from any contamination, thus giving way for carbon oxides, which are the most present potential binding partners in the surrounding atmosphere. That effect might explain the higher carbon content at the Zr1-m surface. Interestingly, despite this carbon increase, an increase in hydrophilicity was recorded on the Zr1-m surface, too. In a study by Hayashi et al. [44], the authors explicitly investigated the adverse biological effects of carbon deposition on the osteoconductivity of titanium, and they reported that the amount of surface carbon plays an important role in the hydrophilicity, settlement, growth and differentiation of osteoblast cells. It was concluded that a correlation between the decrease in surface carbon and the resulting increase in hydrophilicity after UV surface treatment exists. In other words, the less hydrocarbon present on the surface, the more hydrophilic the surface and the better the adhesion and further growth of bone cells. Other study groups also reported UV light-induced photocatalytic removal and direct decomposition of hydrocarbons and carbonaceous species as reasons for the formation of highly wettable titanium and zirconia surfaces [24,32,45,46,47,48]. However, in the present study, the hydrophilicity of the Zr1-m surface increased with a concomitant increase in surface carbon, contradicting the observations of Hayashi et al. [44] and suggesting that the amount of surface carbon alone does not affect the hydrophilicity of surfaces. At this point, however, it must be mentioned as a limitation in our study that our sample number was small. The reasons for this are, on the one hand, Zr2 has a zirconia quality which is used in implants available on the market (e.g., the “Pure” implant from the company Straumann (Switzerland) or the “ceramic.implant” from the company VITA (Germany)). Such implants are intensively tested for their safety in clinical use. The industrial manufacturing process of the material, including surface preparation, is standardized and controlled according to the former Medical Device Directive (MDD). Therefore, we expected that the standard deviation between different samples would be negligible. In a previous study, a monoclinic percentage of 3.0% was measured in a ceramic.implant sample [49], which is very close to the result of the present study and demonstrates the stability of the production process. Therefore, we assumed that one sample was sufficient for Zr2 if multiple measurements were performed on this sample. On the other hand, the other ceramic used, Zr1, was an experimental material of which we unfortunately did not have enough material to perform broader tests.

According to other author groups, surface treatments not only change the chemical composition, but also alter other properties such as the surface energy or the electrostatic potential [50,51], so that detailed analyses of surface energy, electric charge and other physicochemical properties are required to determine the mechanism of osseointegration [4,44]. At this point, the question arises as to how the surface treatment by H_2_O_2_ differs from the UV light-treatment. Compared with the results of previous studies in which the same material surfaces were treated with UV light [24,32], the 15-min UV surface treatment can be regarded as having a greater effect on the surfaces than the two-hour treatment with 20% H_2_O_2_. Hence, UV-treatment removed the abovementioned carbon layer on the Zr1-m surface [24,32], while the H_2_O_2_-treatment, in the dosage and intensity at which it was carried out, was too weak for this purpose and resulted in only superficial cleaning. In this context, it is worth looking at the study by Nagassa et al. [29], in which titanium surfaces were treated with 30% H_2_O_2_ for different lengths of time, and their effects on surface topography, surface chemical composition, hydrophilicity and protein adsorption were investigated. While only minor changes in surface topography/roughness in the nanometer range were noticed after 1–6 h of H_2_O_2_ treatment, a much greater increase in surface roughness in the micrometer range was seen after 24 h and after 1–4 weeks. The maximum increase in oxide thickness and surface chemical change was observed between 1 h and 4 weeks and 3 h and 4 weeks, respectively, although there was no increase in the molar ratio of oxygen to titanium (O1s:Ti2p) or hydrophilicity. Adsorption of plasma and serum albumin increased for 1–24 h on H_2_O_2_-treated discs and continued to increase for 1–4 weeks on H_2_O_2_-treated discs. Therefore, the authors concluded that surface topography/roughness and oxide composition/thickness were more altered by H_2_O_2_-treatment and affected protein adsorption more than hydrophilicity. On the one hand, the results of this study could confirm our previously expressed assumption that a treatment of zirconium surfaces with 20% H_2_O_2_ for only two hours is not sufficient to remove carbons on the surface, and probably higher dosages or treatment periods are necessary to achieve more surface effects. However, a higher concentration of H_2_O_2_ or a longer treatment period appears to be detrimental, as this seems to lead to a greater increase in the monoclinic phase, which in turn is unfavorable for the mechanical stability of the zirconia implant material. [39]. This needs to be investigated in further studies.

On the other hand, the results of the study by Nagassa et al. [29] could also explain why treatment with H_2_O_2_ showed little or no effect on cell behavior in our study. Neither qualitative and quantitative analysis by the immunofluorescence method nor DNA quantification and determination of cell area size after 4 h and 24 h showed that H_2_O_2_-treatment had any effect on osteoblast behavior. Regarding metabolic activity and thus cell growth, a clearly and significantly stronger activity could be observed especially on the untreated and smooth surfaces compared to the H_2_O_2_-treated surfaces. In contrast, the difference between untreated and H_2_O_2_-treated surfaces was less clear on roughened surfaces, so that it can be supposed that there is a surface roughness-dependent effect of H_2_O_2_ on cell proliferation ability.

In terms of the extracellular matrix mineralization capacity of osteoblasts, again no clear effect of H_2_O_2_-treatment on the osteoblast behavior could be observed. Only at two time points could a significantly stronger activity be observed on untreated smooth surface Zr1-m after 4 h and on untreated rough Zr2-r surface at day 14. A similar tendency for the untreated smooth surface Zr2-m can be assumed, although not a significant one. Overall, the tests performed showed no effect of H_2_O_2_-treatment on osteoblast behavior. There is rather little and non-significant evidence that surface texture exerts more influence on cell behavior.

While it is generally accepted that a material with a relatively high surface roughness can accelerate initial cell attachment on roughened implant surfaces compared to smooth surfaces [4,52], one recent study has shown that polished and subsequently heat-treated zirconia surfaces result in better bone cell behavior than equally heat-treated but sandblasted and acid-etched rough surfaces [11]. However, this study used osteoblast-like cell lines (MG-63), which do not fully reflect the behavior of primary human cells. The use of isolated primary human osteoblast cells provides more powerful results and therefore should be the cells of choice used in all areas of in vitro bone biology research [52,53]. However, in that study it is interesting to note that the tetragonal ratio in the zirconia surface increased with heat treatment, while sandblasting resulted in a decrease of the tetragonal phase for the roughened surfaces [11]. In addition, heat treatment improved the wettability of the zirconia, for which the rounder surface morphology was assumed to be a possible explanation [11]. However, the behavior of the cells in that study did not correlate with the wettability of the zirconia surface. Instead, an increased tetragonal phase ratio of the zirconia had a positive effect on the viability of human osteoblasts, while an increased surface roughness of the zirconia decreased cell spreading [11]. In contrast, a study using human osteoblasts showed that rough surfaces favored initial adhesion, while proliferation appeared to improve on smooth surfaces, and gene expression appeared to be more modulated on the smoothest biomaterial [52]. In a clinical study, standardized periapical radiographs over 5 years were used in a prospective two-center cohort study to investigate how the structure and geometry of zirconia implants affect the DIB (distance between implant shoulder and first BIC). Here, the evaluation showed that the peri-implant bone is still stable after 5 years on both rough and smooth sections of the implant. According to this study, the surface (rough or smooth) does not seem to have an influence on long-term osseointegration [54]. It appears that the cellular response on the implant surface is influenced multifactorially, and it is difficult to survey all of them. Indeed, a very recent review criticized the lack of correlation in the results of previous clinical, preclinical and in vitro cell studies on the effects of surface texture of zirconia implants and stated that standardized procedures for human, animal and in vitro studies are needed to clarify the surface aspect [12]. Thus, another review of preclinical data was cited and reported that bone-to-implant contact (BIC) with zirconia implant surfaces depends on the animal model rather than surface roughness [19]. The authors concluded that preclinical data do not indicate a preference for a particular surface texture. Therefore, it seems to remain unknown to this day which surface modification technique leads to the most favorable osseointegration ability. With regard to H_2_O_2_-treatment of zirconia surfaces, further investigation with different concentrations and times of application would be logical in order to find out, on the one hand, to what extent the material quality on the surface changes and, on the other hand, how this affects the cell attractiveness.

## 5. Conclusions

Within the limitations of this study, it can be concluded that exposure of zirconia surfaces to 20% H_2_O_2_ for 2 h increases the wettability of the surfaces, but also seems to increase the monoclinic phase, especially on the roughened surfaces, which can be considered detrimental to material stability. Furthermore, it can be concluded that the performed H_2_O_2_-treatment does not affect cell behavior.

## Figures and Tables

**Table 1:** Atomic percentages of the XPS detail spectra of the smooth and roughened surfaces for the C1s, O1s and Zr3d electrons without and with H_2_O_2_-treatment (arrows pointing upwards indicate an increase, arrows pointing down indicate a decrease in the corresponding elements).

	C1s [at%]	C1s + H_2_O_2_ [at%]	O1s [at%]	O1s + H_2_O_2_ [at%]	Zr3d [at%]	Zr3d + H_2_O_2_ [at%]
**Zr1-m**	34	45 [↑ 32%]	49	42 [↓ 14%]	18	14 [↓ 22%]
**Zr1-r**	30	12 [↓ 60%]	58	73 [↑ 26%]	12	15 [↑ 25%]
**Zr2-m**	36	24 [↓ 33%]	48	53 [↑ 10%]	17	23 [↑ 35%]
**Zr2-r**	28	15 [↓ 46%]	51	61 [↑ 19%]	22	25 [↑ 14%]

**Table 2:** Percentages of the monoclinic phase of all four surfaces before and after H_2_O_2_ treatment.

	Monoclinic Phase (wt%)	
		+H2O2
**Zr1-m**	≈33.6	≈34.4
**Zr1-r**	≈34.5	≈47.9
**Zr2-m**	≈3.4	≈4.0
**Zr2-r**	≈4.9	≈8.3

## Data Availability

Data is contained within the article.
